# Factitious Disorder in Seven Patients: A Saudi Experience

**DOI:** 10.7759/cureus.14135

**Published:** 2021-03-27

**Authors:** Salman S Qasim, Ahmad M Samman, Anas A Alalwan, Omar E Tarabzoni, Enan H Alsharif, Abdulkarim O Alanazi, Laila Layqah, Fares F. Alharbi

**Affiliations:** 1 College of Medicine, King Saud Bin Abdulaziz University for Health Sciences, Riyadh, SAU; 2 Department of Research Office, King Abdullah International Medical Research Center, Riyadh, SAU; 3 Department of Mental Health, Ministry of the National Guard Health Affairs, Riyadh, SAU

**Keywords:** factitious disorder, munchausen syndrome, saudi arabia

## Abstract

Background

Factitious disorder (FD) is a psychiatric disease where signs and symptoms are produced, falsified, or exaggerated consciously in the absence of clear external motivations. Through needless medical visits, costly investigatory testing, and potentially long hospital stays, patients with FD waste valuable time and resources, which affects both the patient and the healthcare system. It can be very challenging for physicians who have never encountered patients with FD to recognize them promptly as symptoms of FD vary greatly.

Methodology

This was a retrospective study of patients diagnosed with FD attending King Abdulaziz Medical City in Riyadh, Saudi Arabia, a tertiary care military hospital and one of the most prominent academic and referral medical institutions in the country. Using the BESTCare health information system, we reviewed patients’ electronic health records from January 2015 to December 2020. The diagnosis of FD was based on the International Classification of Diseases and Related Health Problems 10th edition.

Results

A total of seven patients were included in the study, of whom five (71%) were males and two (29%) were females. Of the total seven patients, three were 21 years old and younger, one was 38 years old, and three were 56 years old and older. Three (43%) patients were married and four (57%) were single. In terms of occupation, three (43%) patients were retired, one (14%) worked in a private company, one (14%) was unemployed, and two (29%) were students. A total of four (57%) patients initially presented to the Emergency Department (ED), and only three (43%) presented to the outpatient clinics. Induced disease or injury was found in five (71%) patients. Induced skin injury was found in four (57%) patients. Counseling and psychotherapy were only offered to one (14%) patient.

Conclusions

FD remains a rare psychiatric condition that is difficult to recognize. Despite a small sample size, FD in the present study showed a male predominance, affecting patients of all age groups. About half of the patients presented initially to the ED. Induced disease or injury was the most commonly reported pattern of clinical presentation. Induced skin injury was the most common clinical presentation found in around half of the patients. We believe that the number of FD patients in the present study is likely underreported and is expected to be higher. This highlights the need for better awareness of FD among physicians in different medical fields. We emphasize that there is a need for better professional training in the identification of FD.

## Introduction

Factitious disorder (FD) or Munchausen syndrome is a psychiatric disease where signs and symptoms are produced, falsified, or exaggerated consciously in the absence of clear external causes or motivations [1]. There are two main types of FD. The first type is FD imposed on self. Patients diagnosed with this type of FD falsify and feign physical and/or psychological symptoms for the sole reason of the emotional satisfaction of appearing ill [1]. For instance, some FD patients can report chest pain that might misguide the physician to believe they are having a heart attack. The second type is FD imposed on another. In this type, the production and falsification of symptoms occur in people under the person’s care such as children, the elderly, disabled individuals, or even pets. The diagnosis in such cases is given to the perpetrator, not the victim [1].

Different epidemiological studies have revealed a wide range of prevalence and incidence rates of FD, which can be attributed to a lack of assessment procedures, detection methods, and clear diagnostic guidelines [2]. A study by Catalina et al. [3] conducted among psychiatric inpatients found that 8% of the patients exhibited factitious symptoms, with symptoms becoming more severe when patients were discharged. Fliege et al. [4] conducted a survey on senior hospital consultants and physicians in private practices, where physicians estimated a one-year prevalence of 1.3% for FD among their patients. The rates reported by dermatologists and neurologists were relatively higher than other specialties. On the other hand, Wallach [5] estimated the base rate of FD at 5% of all physician-patient encounters.

The morbidity and mortality related to FD can be precipitated through one or both of the following: patient’s falsifications of the disease by harming themselves, or the procedures that the medical team performs to manage the patient’s falsified symptoms [6]. Although not fully understood, some risk factors of FD include childhood trauma, personality disorders, depression, and medical employment [7].

It can be very challenging for physicians who have never encountered patients with FD to recognize them promptly as symptoms of FD vary greatly and can include mimicking of a wide range of serious diseases or injuries. Furthermore, patients with FD can continue their deception despite facing objective evidence that does not support their case [7]. Patients with FD, often seeking help from multiple doctors, usually present with vague or inconsistent symptoms that can worsen for no apparent reason, and they do not respond to standard therapies as expected. The main symptoms of FD are consciously induced by the patient with no compensatory intentions. Patients present with false history, exaggerated symptoms, and a mystifying physical examination. Moreover, symptoms of FD can be classified into being predominantly physical, predominantly psychiatric, or combined [8]. Patients with predominantly physical symptoms can present with abdominal pain, chest pain, arthralgia, diarrhea, and many other symptoms. On the other hand, patients with the psychologically predominant subtype can report depression, prolonged bereavement, psychotic thoughts, and suicidal ideations [8]. The diagnosis of FD can be very difficult due to the profound medical knowledge of FD patients. Having a good medical knowledge enables patients with FD to accurately fake various diseases and serious life-threatening conditions. Conducting a detailed interview with the patient and a family member or a friend, reviewing past medical records, and implementing the guidelines of the Diagnostic and Statistical Manual of Mental Disorders may result in an early and proper diagnosis of patients with FD [9].

When it comes to the management of FD, research is lacking [10]. However, a multidisciplinary team approach is recommended when treating FD patients. The team should explain their suspicion to the patient in a supportive manner, focusing on the psychological distress of the patient as a potential source of the illness [11]. Approaches such as cognitive behavioral therapy, psychoanalytic psychotherapy, and supportive therapy have shown modest success in treating FD patients, considering that the majority refuse treatment [12]. In addition, psychiatric assessment should be performed for any comorbid psychiatric disorders and treatment should be provided accordingly.

Due to a lack of local studies highlighting the epidemiological characteristics of patients with factitious disorder, we aimed in this study to assess the demographic features, psychiatric comorbidities, and clinical presentations of patients with FD in a main referral hospital in Riyadh, Saudi Arabia. The findings of this study may assist physicians in the early identification and subsequent treatment of patients with FD.

## Materials and methods

This is a retrospective study of patients diagnosed with FD attending King Abdulaziz Medical City (KAMC) in Riyadh, Saudi Arabia, a tertiary care military hospital. KAMC provides healthcare services for National Guard soldiers and their families with a capacity of 1,501 beds. Healthcare facilities at KAMC include medical and surgical wards, long-term care and rehabilitation wards, antenatal and postpartum wards, intensive care units, trauma and emergency departments, a psychiatric department, an obstetrics and gynecology department, and ambulatory and dental clinics. The medical city also encompasses King Abdullah Specialist Children’s Hospital, a primary referral institute for pediatric patients in Saudi Arabia, along with a specialized cardiac center. Using the BESTCare health information system implemented in our institution in 2015, we reviewed patients’ electronic health records from January 2015 to December 2020. The diagnosis of FD was based on the International Classification of Diseases and Related Health Problems 10th edition (ICD-10).

Patients’ electronic health records were viewed for the extraction of demographic information in terms of gender, age, marital status, and occupation. Presenting department, the pattern of clinical presentation (false report of disease or induced disease), presenting symptoms, psychiatric comorbidities, and history of substance use disorders were also documented.

Prior to data collection, approval by the Institutional Review Board of King Abdullah International Medical Research Center, Ministry of National Guard-Health Affairs, Riyadh, Saudi Arabia was obtained to conduct the present study (approval number RC20/609/R). Patient confidentiality was ensured, and only the study team received and used the patient data. To ensure anonymity, patients medical record numbers were replaced with serial numbers and saved in a separate sheet only accessible to the primary investigator. The requirement for informed consent was waived because of the retrospective nature of the present study and the use of anonymized patient data.

## Results

Of the seven patients, five (71%) were males and two (29%) were females. Patient ages were scattered and ranged between 15 and 60 years. Three patients were 21 years old and younger, one patient was 38 years old, and three patients were 56 years old and older. Of the seven patients, three (43%) were married and four (57%) were single. In terms of occupation, three (43%) patients were retired, one (14%) worked in a private company, one (14%) was unemployed, and two (29%) were students. Although this is a small sample size, more than half of the patients (57%) initially presented to the Emergency Department (ED), and only three (43%) patients presented to the outpatient clinics. Induced disease or injury was the most commonly reported pattern of clinical presentation, which was found in 5 (71%) patients. The remaining 2 (29%) patients falsely reported disease or injury without actively inducing an injury to the body. Induced skin injury was the most common clinical presentation found in four (57%) patients. One (14%) patient had a history of comorbid psychiatric disorder, where he was previously diagnosed with major depressive disorder (MDD). A history of a substance use disorder was observed in two (29%) patients, with the substances abused being benzodiazepine and amphetamine. Counseling and psychotherapy were only offered to one (14%) patient. Detailed patient data are presented in Table 1.

**Table 1:** Demographic and clinical features of patients with factitious disorder. MVA: motor vehicle accident

Patient number	1	2	3	4	5	6	7
Age (years)	18	56	15	60	21	56	38
Gender	Male	Male	Female	Male	Male	Male	Female
Marital status	Single	Married	Single	Married	Single	Married	Single
Occupation	Student	Retired	Student	Retired	Employed	Retired	Unemployed
Presentation department	Emergency department	Emergency department	Outpatient clinic	Outpatient clinic	Emergency department	Outpatient clinic	Emergency department
Pattern of clinical presentation	False report of disease/injury	False report of disease/injury	Induced disease/injury	Induced disease/injury	Induced disease/injury	Induced disease/injury	Induced disease/injury
Presenting symptoms	MVA-related injury	Seizure	Skin lesions	Skin lesions	Skin lesions, abdominal pain	Skin lesions	Fever, cough, vomiting, diarrhea, weakness
History of substance abuse (yes/no)	No	Yes (benzodiazepine)	No	No	Yes (amphetamine)	No	No
Referred for psychotherapy (yes/no)	No	No	No	Yes	No	No	No

## Discussion

FD remains a rare psychiatric condition that may be difficult to recognize. The prevalence of FD varies according to several factors. For example, the type of patient population, study setting, and clinical suspicion and awareness manifested by the healthcare professional can influence the prevalence of this disorder [13]. In the present study, out of the patients attending KAMC from January 2015 to December 2020, only seven patients were assigned the diagnosis of FD. According to previous studies, the prevalence of FD among psychiatry inpatients varied between 0.5 and 8% [13]. We believe that our results showed a relatively low prevalence rate, which may be attributed to a reduced awareness of FD among clinicians and due to the difficulty in the diagnosis of the disorder [7,11,13].

According to Yates and Feldman, in their systematic review of 455 patients diagnosed with FD, more than half of the patients were females [14]. In contrast, our results show a male predominance with nearly three-quarters (71%) of the patients being males. This may be due to KAMC being a military medical institution in which male soldiers are more likely to visit. Moreover, this may have led to clinicians becoming more sensitive towards diagnosing male patients with FD as opposed to females. Furthermore, in the aforementioned systematic review, most patients with FD presented in their early adult life. In the present study, however, most patients did not belong to a specific age group as their ages were scattered and ranged between 15 and 60 years. Due to the small sample size, this may not be a true representation of age distribution among patients diagnosed with FD.

In terms of occupation, research suggests that FD is more common in females who work in healthcare institutions [13,15]. According to Yates and Feldman’s systematic review, nursing was the single most common occupation among patients with FD [14]. They suggested that this may be due to publication bias. The overrepresentation of healthcare professionals in the literature may be a result of them being more capable of using their medical knowledge to feign symptoms more credibly [14]. Another reason that may explain the common reporting of healthcare occupations among patients with FD is the appeal of such occupations for FD patients. Medical procedures, particularly those carried out by nurses, may convey a similar subconscious satisfaction to that of the desire to falsify symptoms and receive medical attention. In the present study, however, no patients reported to have worked or were working in healthcare institutions. In this study, two (29%) patients reported being retired and one (14%) was unemployed. The association between occupation and FD is still not fully understood. This is potentially due to FD being a rare psychiatric disorder and due to the challenges and difficulties physicians encounter in the diagnosis of such cases.

Single patients in our study constituted 57% of the total number of patients with FD, while 43% of the patients were married. A number of studies and reports have suggested similar findings [16-18]. Moreover, the fact that patients with FD are more likely to be unmarried endorses the implication that many of them do not do well in relationships, and hence, are more likely to not get married [19]. In contrast, a systematic review of case reports and series of patients diagnosed with FD showed a predominance of patients who were married. Authors indicated that this finding may be influenced by the lack of information regarding marital status in some cases leading to the true distribution being affected [20]. Marital status was also not addressed in some previously conducted studies [14,21]; hence, there is a necessity to introduce more research investigating the development of FD among married versus single individuals.

Patients diagnosed with FD may present to different departments within the same medical institution. Understanding whether they favor a specific department over another may provide useful information that could aid physicians in the early detection of patients with FD. In the present study, around half of the patients diagnosed with FD initially presented to the ED. This is in agreement with a number of case reports regarding FD [20,22,23]. However, other studies also reported patients presenting to outpatient clinics of different disciplines [14]. The specific department of initial presentation, whether it was ED or outpatient clinics, may give a better insight into the behavior of patients with FD.

The pattern of clinical presentation may be helpful in identifying FD as it is regarded among the first steps before a patient is diagnosed. Upon interviewing, patients with FD would either complain of self-inflicted injuries, falsely report symptoms, or feign a disease or injury with a dramatic but inconsistent medical history [24]. Symptoms or the false reporting of them in patients with FD may vary widely. Examples include induced hypoglycemia, self-inflicted skin injury, diarrhea due to ingestion of laxatives, and many other clinical manifestations. Patients diagnosed with FD deliberately falsify symptoms for the emotional satisfaction of appearing ill without an external gain, such as attention or financial compensation, as seen in malingering [1]. In a systematic review, patients mainly presented with a self-induced injury (58.7%), followed by a false report of symptoms (22.2%), and finally, simulating a disease (19.1%) [14]. In our study, induced disease or injury was the most reported pattern of clinical presentation, which was found in five (71%) patients. The remaining two (29%) patients falsely reported disease or injury without actively inducing an injury to the body. Induced skin injury was the most common clinical presentation found in four (57%) patients. Likewise, another study reported similar findings [24]. The relatively higher number of FD patients manifesting with dermatological symptoms in the present study may be due to dermatologists being more aware about FD. Alternatively, patients with FD might find it fairly easier to feign skin injury compared to other injuries and to draw quick medical attention. Favoring a specific clinical presentation over another is yet to be fully understood and requires more research.

FD often has comorbid psychiatric conditions. The most common of which are depression and personality disorders [14]. Substance use disorder and anxiety have also shown a lower association with FD, yet the reason for this relationship is still unclear. Only one (14%) patient in the present study had MDD. In contrast, the prevalence of depression and personality disorders was found to be around 37% and 43%, respectively, in patients with FD in systemic reviews [20]. This could be due to the fact that not all patients had a full psychiatric assessment done by psychiatrists and comorbidities were missed. Substance use disorder was found in 29% of patients in our study, a higher percentage than the 15% prevalence documented in other reviews [14]. FD patients would often use their ability to convince and deceive physicians to obtain controlled substances [25]; however, the association between substance use disorder and FD has not been well researched and many questions remain unanswered.

The treatment of FD can be very difficult due to factors related to the patient as well as the healthcare professional. Patients with FD require a tremendous amount of compassion, patience, and nonjudgment from a multidisciplinary team to ensure successful treatment [26]. Furthermore, confrontation of patients with FD is usually met with profound emotions of anger, despair, and frustration by both the healthcare professional and the patient [26]. Although psychotherapy is considered the primary treatment of FD [27], its effectiveness and that of other management techniques could not be evaluated due to insufficient evidence [10]. Almost all patients in the present study were not treated for FD, with only their somatic symptoms being managed. The patients either refused psychotherapy at once or were discharged against medical advice. Only one patient was referred for psychiatric follow-up but did not attend any follow-up appointments after his initial discharge. It is not uncommon that patients with FD refuse psychiatric assessment and subsequent treatment because they deny the fact that their illness is caused by themselves, even when confronted with objective evidence [9].

The present study has several limitations. Due to its retrospective nature, psychiatric comorbidities for some patients could not be thoroughly assessed. A complete medical profile for all patients would allow for a better understanding of FD. As the diagnosis of FD is difficult and often goes unrecognized, we believe that its prevalence among the general population may be underestimated, and that the actual number of FD patients could be higher than what is presented in our study. Alternatively, some healthcare professionals may have insufficient knowledge about FD, as reported by Fliege et al. in their survey-based study [4]. Most patients in our study presented with dermatological symptoms. However, this representation may be biased secondary to increased awareness of FD among dermatologists rather than patient’s preference to induce skin injury. Dermatitis artefacta, a self-inflicted dermatologic injury, was added to the ICD several decades before FD, which further makes the diagnosis of FD by dermatologists more likely compared to other specialties [14]. The association between a specific clinical presentation, such as skin injury, and FD is yet to be fully understood and requires more research. Lastly, although diagnosed with FD, the possibility of a misdiagnosis in some patients cannot be completely ruled out.

## Conclusions

FD remains a rare psychiatric condition that is difficult to recognize. Despite a small sample size, FD in the present study showed a male predominance, affecting patients of all age groups. About half of the patients presented initially to the ED. Induced disease or injury was the most commonly reported pattern of clinical presentation. Induced skin injury was the most common clinical presentation found in around half of the patients. We believe that the number of FD patients in the present study is likely underreported and is expected to be higher. This highlights the need for a better awareness of FD among physicians in different medical fields. We emphasize that there is a need for better professional training in the identification of FD, which may prevent devastating outcomes of this disorder and render physicians more likely to refer patients to psychiatrists for psychotherapy. Further research is required to better understand the relationship between FD and gender, occupation, and marital status.
